# Circulating heat shock protein 27 as a novel marker of subclinical atherosclerosis in type 2 diabetes: a cross-sectional community-based study

**DOI:** 10.1186/s12872-020-01456-7

**Published:** 2020-04-25

**Authors:** Xinru Wang, Jie Shi, Bin Lu, Weiwei Zhang, Yehong Yang, Jie Wen, Renming Hu, Zhen Yang, Xuanchun Wang

**Affiliations:** 1 Department of Endocrinology, Huashan Hospital, Fudan University, 12 Wulumuqi Zhong Road, Shanghai, 200040 China; 2 Department of Endocrinology, Xinhua Hospital, Shanghai Jiaotong University School of Medicine, 1665 Kongjiang Road, Shanghai, 200092 China

**Keywords:** HSP27, Carotid intima–media thickness, Subclinical atherosclerosis, Type 2 diabetes

## Abstract

**Background:**

Heat shock protein 27 (HSP27) has been proposed as a vital protective factor in atherosclerosis. The objective of the present study was to evaluate the association between circulating HSP27 and carotid intima–media thickness (IMT) in individuals with type 2 diabetes and to determine whether HSP27 represents an independent marker of subclinical atherosclerosis in this patient population.

**Methods:**

We performed a cross-sectional community-based study in 186 Chinese subjects with a median duration of type 2 diabetes of 8.2 years who underwent ultrasound carotid IMT measurement. Serum HSP27 levels were assessed by ELISA.

**Results:**

Serum HSP27 levels were significantly higher in the IMT (+, > 1.0 mm) group than in the IMT (−, ≤1.0 mm) group, with the median values of 8.80 ng/mL (5.62–12.25) and 6.93 ng/mL (4.23–9.60), respectively (*P* = 0.006). The discriminative value of HSP27 to evaluate IMT was 7.16 ng/mL and the area under the curve was 0.72 (95%CI = 0.64–0.80, *P* = 0.0065). Spearman’s rank correlation analysis demonstrated that the concentrations of circulating HSP27 were positively associated with carotid IMT (*r* = 0.198, *P* = 0.007) and blood urea nitrogen (*r* = 0.170, *P* < 0.05). Furthermore, in the logistic model, serum HSP27 levels were found to be independent predictors for carotid IMT in type 2 diabetic patients after adjustment for onset age of diabetes, blood pressure, total cholesterol and C-reactive protein (OR = 1.085, *P* = 0.022).

**Conclusions:**

Circulating HSP27, positively correlates with carotid IMT, is an independent predictor for early atherosclerotic changes in diabetes, and may represent a novel marker of subclinical atherosclerosis in type 2 diabetes.

## Background

Atherosclerotic cardiovascular disease (CVD) is the dominating cause of increasing mortality among patients with type 2 diabetes. Increasing carotid intima–media thickness (IMT), closely associated with CVD, is generally accepted as a surrogate marker of atherosclerosis [1–3].

Heat shock protein 27 (HSP27), also termed HSPB1 [4], is a ubiquitously expressed member of the small heat shock protein family [5]. Originally identified as an intracellular molecular chaperone, HSP27 facilitates the correct folding of proteins [6]. In recent years, the extracellular effects of HSP27 on the cardiovascular system have indicated a protective effect against atherosclerosis [7]. Clinical studies have reported that serum HSP27 levels were dramatically decreased in patients with carotid atherosclerosis compared with healthy controls [8]. Low circulating HSP27 levels were found to be associated with high risk of coronary artery disease [9]. Furthermore, reduced HSP27 levels were observed in unstable plaques versus stable plaques [10].

Although these studies have implicated certain roles of HSP27 in atherosclerosis or coronary heart disease (CHD), little is known about the direct relationship between serum HSP27 concentration and subclinical atherosclerosis in the context of type 2 diabetes. In the present study, we examined the association between circulating HSP27 levels and carotid IMT in patients with type 2 diabetes to determine whether HSP27 may represent a potential predictor for early-stage atherosclerosis in this patient population.

## Methods

### Participants and study design

This trial was designed as a cross-sectional study. Participants were recruited in Shanghai from February 2004 to July 2004. Twenty residential areas administered by 20 residents’ committees were sampled randomly in the central area of Shanghai. Questionnaires administered by endocrinologists and primary care clinicians were used to identify individuals with type 2 diabetes. Two hundred individuals were randomly selected, and 186 of them with complete information were enrolled in our study [11]. Based on carotid IMT values detected by color ultrasound, subjects were stratified into an IMT (−) group (*n* = 110) or an IMT (+) group (*n* = 76). The IMT (−) group included patients with carotid IMT levels ≤1.0 mm while the IMT (+) group contained patients with IMT values > 1.0 mm. All subjects provided written informed consent prior to participation. The research protocol was approved by the Institutional Review Board of Huashan Hospital, Fudan University School of Medicine.

### Anthropometric parameters and biochemical indexes

Detailed histories of all the subjects were obtained using questionnaires. Physical examination and anthropometric measurements were performed by trained physicians. Waist circumference (WC) was measured at the midpoint between the lower margin of the least palpable rib and the top of the iliac crest in the late exhalation phase in a standing position. Hip circumference was measured around the widest portion of the buttocks using a tape measure parallel to the floor. Blood pressure measurement was obtained using a standard manual mercury sphygmomanometer at a steady state on the upper arm. Venous blood samples were collected between 7:00 and 8:00 AM from the antecubital vein of each subject after overnight fasting. Blood glucose, serum insulin, total cholesterol (TC), triglycerides (TG), high-density lipoprotein cholesterol (HDL-C), low-density lipoprotein cholesterol (LDL-C), blood urea nitrogen (BUN), and serum creatinine (Scr) were determined using standard methods in a qualified laboratory with a Hitachi 7080 analyzer (Hitachi, Ltd., Tokyo, Japan). C-reactive protein (CRP) was measured in duplicate by ELISA using a Duoset kit (DY1707, R&D Systems, Minneapolis, MN). Glycated hemoglobin A1c (HbA1c) was assessed by high-pressure liquid chromatography (HLC-723G7; Tosoh, Shanghai, China). The homeostasis model assessment of insulin resistance (HOMA-IR) was calculated based on the formula of Matthews et al. [12]. The MDRD Study equation was used for the calculation of estimated glomerular filtration rate (eGFR) [13].

Type 2 diabetes was defined as the presence of ≥1 of the following criteria: fasting plasma glucose ≥ 7.0 mmol/L; plasma glucose ≥11.1 mmol/L 2 h after a 75-g oral glucose load as in a glucose tolerance test (OGTT); and symptoms of high blood sugar and casual plasma glucose ≥11.1 mmol/L. CVD was defined as stroke and CHD, including unstable or stable angina and myocardial infarction, while lipid-lowering drugs referred to statins or fibrates.

### Measurement of serum HSP27

Serum HSP27 levels were measured in duplicate using a commercial enzyme-linked immunosorbent assay kit (QIA119, Calbiochem, San Diego, CA) according to the manufacturer’s instructions. The intra- and inter-assay coefficients of variation of the ELISA were 6.2 and 8.3%, respectively.

### Measurement of carotid IMT

With reference to the European Mannheim carotid IMT consensus, the intima–media thickness values of the common carotid arteries of the subjects were measured using an Acuson Sequoia 512 system (Siemens Medical Solutions USA, Mountain View, CA). The procedure was performed in subjects in a supine position by an experienced ultra-sonographer who was unaware of the subjects’ demographic and clinical characteristics. Three arterial sites were evaluated: the bilateral distal common carotid arteries, the carotid bulbs, and the proximal internal carotid arteries. Different scanning angles (anterior, lateral, posterior) were used to identify the thickest IMT in each wall. Both left and right carotid IMTs were assessed, and three measurements were performed for each subject. The mean values of the maximum IMT in both left and right sides of the common carotid arteries were defined as carotid artery IMT.

### Statistical analysis

Quantitative data were evaluated using the Kolmogorov–Smirnov test to determine whether they followed a normal distribution. Parameters were considered normally distributed if *P* > 0.05. Normally distributed data were reported as means and standard deviations, while variables with a skewed distribution were expressed as medians (interquartile range). Categorical variables were presented as frequencies and percentages. One-way ANOVA and a Chi-squared test were used for comparisons between the two groups. Spearman’s correlation was used to evaluate the correlation between serum HSP27 concentration and other clinical indexes. Receiver operating characteristics (ROC) curve analysis was performed to calculate the area under the curve (AUC) and the cutoff value of HSP27 for IMT. The determinants of carotid IMT were explored using univariate and multivariate logistic analysis. Linear regression analysis was also used. Variables with statistically significant correlations (*P* < 0.05) in univariate analysis were examined in the multivariate model. All statistical analyses were conducted using the SPSS version 25 (IBM Corp., Armonk, NY) and Prism 8 software (GraphPad, San Diego, CA). Two-sided values of *P* < 0.05 were considered statistically significant.

## Results

### Subject characteristics

The characteristics of the 186 subjects and the two subgroups divided according to carotid IMT (1.0 mm) are shown in Table 1. The low median HbA1c (6.70%) and normal eGFR (121.15 ± 30.98 mL·min^− 1^ 1.73 m^− 2^) values were indicative of generally well-controlled type 2 diabetes among the study subjects. The mean IMT levels in the IMT (−) and IMT (+) groups were 0.78 mm and 1.41 mm, respectively. Compared with the IMT (−) individuals, the IMT (+) group was 6 years older (*P* < 0.001) and had a higher onset age of diabetes (*P* < 0.01). The levels of systolic blood pressure (SBP) (*P* < 0.05), diastolic blood pressure (DBP) (*P* < 0.05), TC (*P* < 0.05), and CRP (*P* < 0.05) were significantly higher in the IMT (+) group than in the IMT(−) group. There were no statistical differences in sex, smoking status, alcohol consumption, use of lipid-lowering drugs, history of CVD, duration of type 2 diabetes, BMI, WC, waist–hip ratio (WHR), FBG, postprandial blood glucose (PBG), fasting insulin, fasting C peptide, 2 h insulin, 2 h C peptide, HOMA-IR, HbA1c, BUN, Scr, serum uric acid, eGFR, TG, LDL-C, HDL-C between the two groups (all *P* > 0.05, Table 1).

**Table 1:** General characteristics of study subjects

Characteristic	Total	IMT (−)	IMT (+)	*P* value
Subjects	186	110	76	–
Age (years)	67 (57–73)	64 (53.75–71)	70 (64.25–75)	0.000*
Sex (male/female)	74/112	39/71	35/41	0.147
Smoking (%)	76.88	78.18	75.00	0.613
Alcohol (%)	8.60	9.09	7.89	0.078
lipid-lowering drugs (%)	50.98	60.91	51.32	0.194
CVD (%)	20.43	17.27	25.00	0.199
Onset age of diabetes (years)	57.12 ± 11.16	54.76 ± 11.29	60.47 ± 10.13	0.001*
Duration of diabetes (years)	6 (3–10)	6 (3–10)	7 (4–12)	0.370
BMI (kg/m^2^)	24.63 ± 3.21	24.46 ± 3.19	24.88 ± 3.23	0.392
WC (cm)	81.43 ± 8.76	81.10 ± 8.75	81.90 ± 8.81	0.544
WHR	0.87 ± 0.06	0.87 ± 0.07	0.88 ± 0.06	0.077
SBP (mmHg)	140 (130–150)	140 (129.5–150)	142 (135–160)	0.001*
DBP (mmHg)	84 (78–92)	82 (76–90)	86 (80–94.75)	0.026*
FBG (mmol/L)	7.80 (6.45–10.00)	7.55 (6.3–9.65)	8.60 (6.90–10.10)	0.236
PBG (mmol/L)	15.73 ± 6.08	15.60 ± 6.16	15.93 ± 6.00	0.717
Fasting insulin (pmol/L)	12.20 (7.44–20.06)	12.22 (7.35–18.81)	12.20 (7.54–21.77)	0.387
2 h insulin (pmol/L)	46.49 (28.06–69.77)	47.46 (29.43–70.23)	43.99 (25.77–69.63)	0.780
Fasting C peptide (ng/mL)	2.94 (2.22–3.78)	2.96 (2.23–3.72)	2.91 (2.15–3.86)	0.622
2 h C peptide (ng/mL)	8.88 (6.61–11.74)	8.84 (7.07–10.92)	8.88 (6.45–12.67)	0.191
HOMA-IR	4.56 (2.39–7.99)	4.11 (2.23–7.42)	5.27 (2.72–9.22)	0.359
HbA1c (%)	6.70 (6.00–7.65)	6.70 (6.00–7.65)	6.80 (6.03–7.75)	0.730
BUN (mmol/L)	6.00 (5.00–7.15)	5.85 (5.00–6.70)	6.20 (5.20–7.70)	0.224
Scr (μmol/L)	65 (54–78)	62 (52–76)	68 (58–80)	0.441
Serum uric acid (μmol/L)	0.27 (0.24–0.33)	0.28 (0.23–0.33)	0.27 (0.24–0.33)	0.887
eGFR (mL·min^−1^ 1.73 m^−2^)	121.15 ± 30.98	124.15 ± 31.80	116.74 ± 29.41	0.111
TC (mmol/L)	5.26 ± 1.18	5.07 ± 0.99	5.54 ± 1.38	0.008*
TG (mmol/L)	1.60 (1.07–2.16)	1.50 (1.09–2.11)	1.63 (1.01–2.28)	0.482
LDL-C (mmol/L)	3.00 (2.40–3.40)	2.90 (2.40–3.30)	3.00 (2.40–3.60)	0.094
HDL-C (mmol/L)	1.30 (1.05–1.50)	1.20 (1.00–1.50)	1.30 (1.10–1.60)	0.266
UACR (μg/mg)	14.42 (6.04–31.06)	11.29 (4.59–24.94)	20.17 (7.38–46.28)	0.651
CRP (mg/L)	3.05 (1.65–5.29)	6.92 (4.23–9.60)	8.80 (5.62–12.25)	0.019*
IMT (mm)	1.03 ± 0.42	0.78 ± 0.19	1.41 ± 0.38	0.000*
HSP27 (ng/mL)	7.85 (4.78–10.92)	6.93 (4.23–9.60)	8.80 (5.62–12.25)	0.007*

*P* < 0.05 (*)

### Difference in serum HSP27 between the two groups

**Fig. 1:**
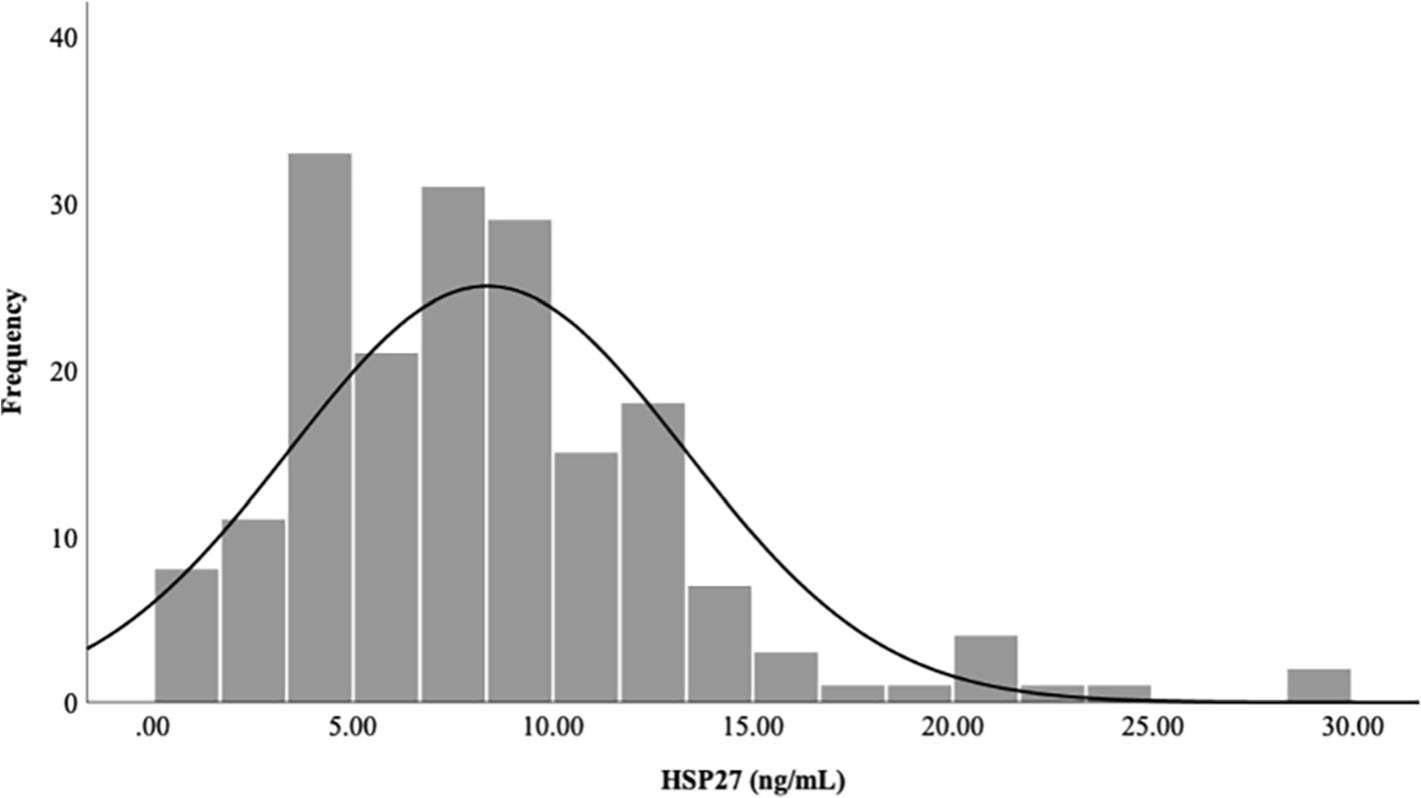
Distribution of serum HSP27 levels in the participants

**Fig. 2:**
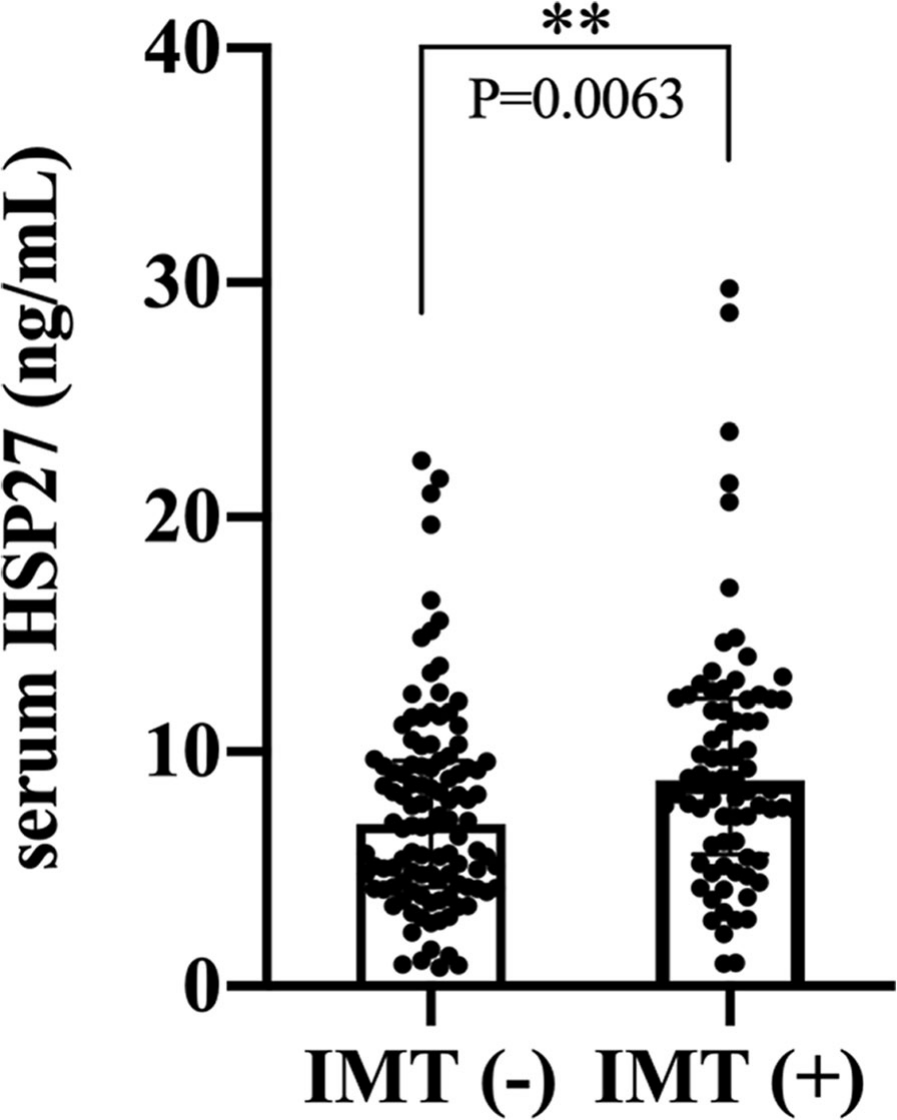
Serum HSP27 in the IMT (−) and IMT (+) subjects (divided according to the values of IMT 1.0 mm). Data are expressed as median (IQR), *P*<0.01(**)

The median value of serum HSP27 in the study population was 7.85 ng/mL (IQR: 4.78–10.92, Fig. 1). As shown in Fig. 2, the median serum HSP27 level in the IMT (+) group was 8.80 ng/mL (IQR: 5.62–12.25, Table 1), significantly higher than that in the IMT (−) group (6.93 ng/mL, IQR: 4.23–9.60, *P* = 0.006).

### Association between serum HSP27 level and carotid IMT

Logistic regression analysis was used to identify independent determinants of carotid IMT. Seven factors were identified as significant predictors using univariate analysis, including age, onset age of diabetes, SBP, DBP, TC, CRP, and HSP27 (all *P* < 0.05, Table 2). In the multivariate model, these factors were defined as covariates, while carotid IMT (≤1.0 mm or > 1.0 mm) was introduced as a dependent variable. After adjusting for onset age of diabetes, SBP, DBP, TC and CRP, the results illustrated that age and HSP27 were still independently associated with carotid IMT in type 2 diabetes, with adjusted odds ratios of 1.061 (*P* = 0.028) and 1.085 (*P* = 0.022), respectively (Table 3). Moreover, a positive relationship between HSP27 and carotid IMT (β = 0.019, *P* = 0.002) was also observed in the multiple linear regression model (Supplemental Table 1 and Table 2). Age (β = 0.190, *P* = 0.007) and TC (β = 0.081, *P* = 0.001) independently correlated with carotid IMT in multiple linear regression analysis.

**Table 2:** Univariate logistic analysis: predictors for carotid artery IMT

	B	OR	95%CI		*P* value
			Lower	Upper	
Sex	−0.441	0.643	0.354	1.169	0.148
Age	0.067	1.069	1.035	1.105	0.000*
Onset age	0.050	1.053	1.021	1.083	0.001*
Duration of diabetes	0.019	1.019	0.978	1.062	0.370
Smoking	−0.178	0.837	0.420	1.667	0.613
Alcohol	0.154	1.167	0.405	3.358	0.775
Lipid-lowering drugs	0.391	1.478	0.819	2.669	0.195
CVD	−0.468	0.626	0.306	1.283	0.201
BMI	0.040	1.041	0.950	1.141	0.390
WC	0.010	1.011	0.977	1.045	0.542
WHR	4.345	77.115	0.605	9837.212	0.079
SBP	0.029	1.029	1.011	1.048	0.002*
DBP	0.032	1.033	1.003	1.063	0.028*
FBG	0.055	1.056	0.964	1.157	0.239
PBG	0.009	1.009	0.961	1.059	0.716
HbA1c	0.033	1.034	0.857	1.246	0.729
Fasting insulin	0.005	1.005	0.994	1.016	0.403
2 h insulin	0.001	1.001	0.994	1.007	0.779
fasting C peptide	0.056	1.058	0.847	1.322	0.620
2 h C peptide	0.026	1.027	0.986	1.069	0.207
HOMA-IR	0.014	1.014	0.984	1.044	0.368
BUN	0.092	1.097	0.944	1.275	0.227
Scr	0.004	1.004	0.993	1.016	0.448
Serum uric acid	0.310	1.364	0.020	94.590	0.886
eGFR	−0.008	0.992	0.982	1.002	0.113
TC	0.349	1.417	1.081	1.858	0.012*
TG	− 0.076	0.927	0.750	1.146	0.485
LDL-C	0.306	1.358	0.944	1.955	0.099
HDL-C	0.409	1.505	0.733	3.089	0.265
CRP	0.099	1.105	1.011	1.207	0.028*
UACR	0.000	1.000	0.998	1.001	0.655
HSP27	0.084	1.088	1.021	1.159	0.009*

*P* < 0.05 (*)

**Table 3:** Multiple logistic regression analysis: independent predictors for carotid IMT in type 2 diabetes

	B	OR	95%CI		*P* value
			Lower	Upper	
Age	0.059	1.061	1.006	1.119	0.028*
Onset age	0.005	1.005	0.958	1.053	0.851
SBP	0.004	1.004	0.977	1.031	0.795
DBP	0.036	1.037	0.994	1.081	0.091
TC	0.260	1.297	0.964	1.744	0.086
CRP	0.051	1.052	0.963	1.149	0.259
HSP27	0.081	1.085	1.012	1.163	0.022*

### ROC curve analysis

**Fig. 3:**
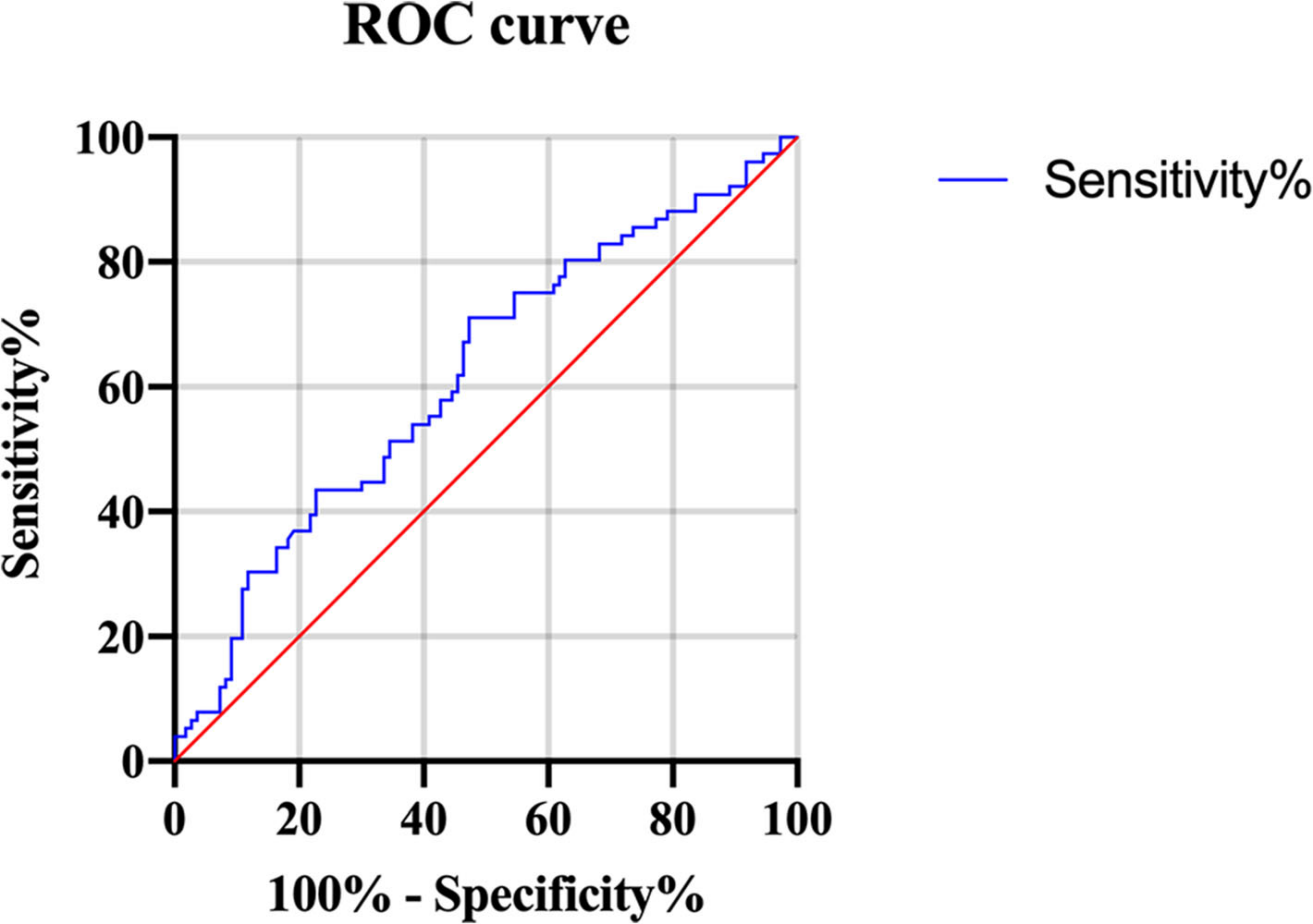
The receiver operating characteristic (ROC) curve of HSP27 in patients with type 2 diabetes with > 1 and < 1 carotid IMT. The area under the curve was 0.72 (95%CI = 0.64–0.80, *P* = 0.0065)

ROC curve analysis was applied to identify an optimal cutoff value for HSP27 to discriminate between > 1 and < 1 IMT (Fig. 3). The AUC of the ROC curve for predicting IMT was 0.72 (95%CI = 0.64–0.80, *P* = 0.0065) and the optimal cutoff value of HSP27 was 7.16 ng/mL with sensitivity of 75.05% (95% CI =64.04–84.04) and low specificity of 67.73% (95% CI =58.46–76.81).

### Correlations between serum HSP27 and other clinical parameters

As presented in Table 4, serum HSP27 levels of the subjects correlated positively with BUN (*r* = 0.170, *P* < 0.05) and carotid IMT (*r* = 0.198, *P* = 0.007), whereas the relationships between HSP27 and other clinical parameters were not significant.

**Table 4:** Correlations between serum HSP27 and other parameters in type 2 diabetes

variable	HSP27	
	*r*	*P* value
Age	0.074	0.313
Onset age	0.091	0.217
Duration of diabetes	0.000	0.999
BMI	0.084	0.253
WC	−0.005	0.949
WHR	0.023	0.753
SBP	0.041	0.578
DBP	0.018	0.811
FBG	0.08	0.281
PBG	0.005	0.945
HbA1c	0.097	0.186
Fasting insulin	−0.142	0.054
2 h insulin	− 0.065	0.383
Fasting C peptide	0.033	0.659
2 h C peptide	0.072	0.332
HOMA-IR	−0.083	0.262
BUN	0.170	0.020*
Scr	0.06	0.414
Serum uric acid	−0.075	0.312
eGFR	−0.018	0.812
TC	0.053	0.474
TG	0.035	0.637
LDL-C	0.092	0.215
HDL-C	0.070	0.341
CRP	0.048	0.519
UACR	0.098	0.182
carotid IMT	0.198	0.007*

*r*: Spearman’s correlation coefficient. *P* < 0.05 (*)

## Discussion

The major findings of this study were that the median HSP27 level in the IMT (+) group was significantly higher than that in the IMT (−) group, and serum HSP27 level positively correlated with carotid IMT (*r* = 0.198, *P* = 0.007). The lack of circulating biomarkers of early-stage atherosclerosis in diabetes requires further clinical investigation. We identified that HSP27 was an independent predictor for subclinical atherosclerosis in patients with type 2 diabetes, even after adjusting for several clinical factors. Therefore, our findings may support the diagnostic value of elevated circulating HSP27. Furthermore, we also found that serum HSP27 concentrations were positively associated with BUN but not with other clinical parameters, which is in alignment with a previous study that showed a correlation between HSP27 and serum creatinine level [14].

HSP27 acts as an antioxidant with the ability to reduce the levels of reactive oxygen species (ROS) through increased intracellular glutathione and decreased intracellular iron [15], which indicates a potential atheroprotective role of HSP27 in atherosclerosis. Another mechanism by which HSP27 exerts its protective function may be attributed to its binding to scavenger receptor-A (SR-A), thus leading to the prevention of acetylated low-density lipoprotein (acLDL) uptake and attenuation of foam cell formation [16]. HSP27 may also reduce the cholesterol content in plaques by more than 30% [17]. In apolipoprotein E null (ApoE^−/−^) mice, which are prone to atherosclerosis, extracellular HSP27 was reported to activate the NF-κB signaling pathway to induce increased expression of granulocyte-monocyte colony-stimulating factor (GM-CSF), ATP-binding cassette transporter A1 (ABCA1), and ATP-binding cassette transporter G1 (ABCG1), thus facilitating cholesterol efflux [17]. In addition, overexpression of HSP27 in this mouse model contributed to a reduction in lesion formation and plaque stability [9, 16, 18]. Stimulation of the release of anti-inflammatory IL-10 via the p38 signaling pathway may also contribute to this effect [19].

An immediate response to stress, inflammation, and cellular damage is to secrete HSP27 into the blood to protect the body [20]. Given the protective role of HSP27 in protecting vessels from oxidative stress [21] and inhibiting inflammation [22], the higher levels of serum HSP27 observed in the IMT (+) group compared with the IMT (−) group in our study may represent a consequence of a compensatory response to inflammation and oxidative stress in the early stage of atherosclerosis. HSP27 may have been induced to counteract these unfavorable factors in the initiation of atherosclerosis. Similarly, Park et al. reported that circulating HSP27 levels in acute coronary syndrome (ACS) patients were remarkably higher than those in an age- and sex-matched healthy controls [23]. Compared with controls, a significant increase in serum HSP27 in patients with CHD was also observed in a recent study [24].

Contrary to these results, circulating HSP27 levels were reported as decreased > 70% in patients with carotid stenosis [8] and coronary artery diseases (CAD) compared with healthy subjects [25]. In addition to reduced concentrations of HSP27, patients with CAD in these two studies had more severe comorbidities compared with patients having higher HSP27 levels in the studies of Park et al. and Zhang et al. [23, 24], including diabetes and hypertension in both studies and smoking in the former plus hyperlipemia in the latter. Patients in these two studies even had serum HSP27 levels decreased to mean values of 0.19 ng/mL and 1.23 ng/mL, respectively. Therefore, we speculated that the condition of the patients in the two studies was too severe to allow sufficient HSP27 secretion to compensate for inflammation and oxidative stress associated with the reported comorbidities. In addition, the degradation of extracellular HSP27 by proteases such as upregulated plasmin in plaques [26] and matrix metalloproteases (MMPs) [27, 28] may also have accounted for the decline in serum HSP27.

Our study had several limitations. First, this cross-sectional study was confined to a specific time point and was therefore unable to identify cause-effect relationships. Given the lack of follow-up data, whether HSP27 was an independent marker of future cardiovascular events remained to be explored. Second, our study included a small number of subjects. Finally, although we have identified a relationship between circulating HSP27 and carotid IMT in type 2 diabetes, the underlying mechanisms of the direct role of HSP27 remains unclear.

## Conclusions

Our study findings demonstrate a positive correlation between circulating HSP27 and carotid IMT, indicating that serum HSP27 may represent a novel biomarker of the progression and diagnosis of subclinical atherosclerosis in type 2 diabetes.

## Supplementary information

**Additional file 1: Table S1.** Univariate linear analysis for variables associated with carotid IMT. **Table S2.** Multiple stepwise regression analysis showing independent predictors for carotid IMT in type 2 diabetes.

## Data Availability

Datasets are available from the corresponding author upon reasonable request.

## Abbreviations

IMT: Intima–media thickness
CVD: Cardiovascular disease
BMI: Body mass index
WC: Waist circumference
WHR: Waist–hip ratio
SBP: Systolic blood pressure
DBP: Diastolic blood pressure
FBG: Fasting blood glucose
PBG: Postprandial blood glucose
HOMA-IR: Homeostatic model assessment of insulin resistance
HbA1c: Glycated hemoglobin A1c
BUN: Blood urea nitrogen
Scr: Serum creatinine
eGFR: Estimated glomerular filtration rate
TC: Total cholesterol
TG: Triglycerides
LDL-C: Low density lipoprotein-cholesterol
HDL-C: High density lipoprotein-cholesterol
CRP: C-reactive protein
UACR: Urine albumin-to-creatinine ratio
OR: Odds ratio
CI: Confidence interval
