# Supplementary material for: Circulating heat shock protein 27 as a novel marker of subclinical atherosclerosis in type 2 diabetes: a cross-sectional community-based study
Source: BMC Cardiovasc Disord. 2020 Apr 25;20:198. doi: 10.1186/s12872-020-01456-7 (PMC7183629; doi:10.1186/s12872-020-01456-7)
Supplement: Supplementary file 1 — Additional file 1: Table S1. Univariate linear analysis for variables associated with carotid IMT. Table S2. Multiple stepwise regression analysis showing independent predictors for carotid IMT in type 2 diabetes. [file 12872_2020_1456_MOESM1_ESM.docx]

**Supplemental Table 1**

| Table 1. Univariate linear analysis for variables associated with carotid IMT | | | |
| --- | --- | --- | --- |
| Variables | Unstandardized | Standardized Coefficients | *P* value |
|  | B | B |  |
| Sex | -0.060 | -0.069 | 0.346 |
| Age | 0.010 | 0.252 | 0.001* |
| Onset age | 0.008 | 0.213 | 0.004* |
| Duration of diabetes | 0.002 | 0.031 | 0.678 |
| Smoking | 0.063 | 0.063 | 0.396 |
| alcohol | 0.009 | 0.006 | 0.935 |
| lipid-lowering drugs | 0.003 | 0.004 | 0.962 |
| CVD | 0.104 | 0.099 | 0.177 |
| BMI | 0.011 | 0.085 | 0.250 |
| WC | -0.001 | -0.019 | 0.800 |
| WHR | 0.289 | 0.042 | 0.567 |
| SBP | 0.005 | 0.224 | 0.002* |
| DBP | 0.005 | 0.118 | 0.108 |
| FBG | 0.011 | 0.084 | 0.255 |
| PBG | 0.003 | 0.042 | 0.574 |
| HbA1c | 0.016 | 0.060 | 0.412 |
| Fasting insulin | 0.000 | 0.012 | 0.872 |
| 2h insulin | 0.001 | 0.079 | 0.287 |
| Fasting C peptide | 0.035 | 0.109 | 0.141 |
| 2h C peptide | 0.01 | 0.178 | 0.015* |
| HOMA-IR | 0.001 | 0.035 | 0.636 |
| BUN | 0.018 | 0.086 | 0.246 |
| Scr | 0.001 | 0.079 | 0.288 |
| Serum uric acid | 0.448 | 0.073 | 0.322 |
| eGFR | -0.002 | -0.125 | 0.089 |
| TC | 0.096 | 0.266 | 0.000* |
| TG | -0.002 | -0.008 | 0.912 |
| LDL-C | 0.104 | 0.203 | 0.006* |
| HDL-C | 0.037 | 0.035 | 0.634 |
| CRP | 0.014 | 0.173 | 0.018* |
| UACR | 3.297E-5 | 0.019 | 0.800 |
| HSP27 | 0.021 | 0.248 | 0.001* |
| *P*＜0.05 (*) | | | |

**Supplemental Table 2**

| Table 2. Multiple stepwise regression analysis showing independent predictors for carotid IMT in type 2 diabetes | | | | |
| --- | --- | --- | --- | --- |
| Variables | Unstandardized | Standardized |  | *P* value |
|  | β | β | t |  |
| Age | 0.008 | 0.190 | 2.727 | 0.007 |
| TC | 0.081 | 0.227 | 3.260 | 0.001 |
| HSP27 | 0.019 | 0.217 | 3.134 | 0.002 |
